# Depression, anxiety and PTSD symptoms before and during the COVID-19 pandemic in the UK

**DOI:** 10.1017/S0033291722002501

**Published:** 2023-09

**Authors:** K. S. Young, K. L. Purves, C. Hübel, M. R. Davies, K. N. Thompson, S. Bristow, G. Krebs, A. Danese, C. Hirsch, C. E. Parsons, E. Vassos, B. N. Adey, S. Bright, L. Hegemann, Y. T. Lee, G. Kalsi, D. Monssen, J. Mundy, A. J. Peel, C. Rayner, H. C. Rogers, A. ter Kuile, C. Ward, K. York, Y. Lin, A. B. Palmos, U. Schmidt, D. Veale, T. R. Nicholson, T. A. Pollak, S. A. M. Stevelink, T. Moukhtarian, A. R. Martineau, H. Holt, B. Maughan, A. Al-Chalabi, K. Ray Chaudhuri, M. P. Richardson, J. R. Bradley, P. F. Chinnery, N. Kingston, S. Papadia, K. E. Stirrups, R. Linger, M. Hotopf, T. C. Eley, G. Breen

**Affiliations:** 1 Social, Genetic and Developmental Psychiatry Centre, Institute of Psychiatry, Psychology & Neuroscience, King's College London, London SE5 8AF, UK; 2 NIHR Maudsley Biomedical Research Centre, King's College London, London, UK; 3 Department of Economics and Business Economics, National Centre for Register-based Research, Aarhus University, Aarhus, Denmark; 4 Department of Child & Adolescent Psychiatry, Institute of Psychiatry, Psychology & Neuroscience, King's College London, London SE5 8AF, UK; 5 National and Specialist CAMHS Trauma, Anxiety, and Depression Clinic, South London and Maudsley NHS Foundation Trust, London, UK; 6 Department of Psychology, Institute of Psychiatry, Psychology and Neuroscience, King's College London, London, UK; 7 Interacting Minds Center, Department of Clinical Medicine, Aarhus University, Aarhus, Denmark; 8 Department of Psychological Medicine, Institute of Psychiatry, Psychology & Neuroscience, King's College London, London, UK; 9 South London and Maudsley NHS Foundation Trust, London, UK; 10 Section of Neuropsychiatry, Department of Psychosis Studies, Institute of Psychiatry, Psychology and Neuroscience, King's College London, London, UK; 11 Blizard Institute, Barts and The London School of Medicine and Dentistry, Queen Mary University of London, London E1 2AT, UK; 12 Department of Basic and Clinical Neuroscience, Institute of Psychiatry, Psychology and Neuroscience, King's College London, London, UK; 13 Parkinson Foundation Centre of Excellence, King's College and King's College Hospital, London, UK; 14 NIHR BioResource and NIHR Cambridge Biomedical Research Centre, Cambridge University Hospitals NHS Foundation, Cambridge Biomedical Campus, Cambridge CB2 0QQ, UK; 15 Department of Clinical Neurosciences and MRC Mitochondrial Biology Unit, University of Cambridge, Cambridge Biomedical Campus, Cambridge CB2 0QQ, UK; 16 Department of Haematology, University of Cambridge, Cambridge Biomedical Campus, Cambridge, UK; 17 Department of Public Health and Primary Care, University of Cambridge, Cambridge Biomedical Campus, Cambridge CB2 0QQ, UK

**Keywords:** Anxiety, COVID-19, depression, PTSD

## Abstract

**Background:**

The impact of the coronavirus disease 2019 (COVID-19) pandemic on mental health is still being unravelled. It is important to identify which individuals are at greatest risk of worsening symptoms. This study aimed to examine changes in depression, anxiety and post-traumatic stress disorder (PTSD) symptoms using prospective and retrospective symptom change assessments, and to find and examine the effect of key risk factors.

**Method:**

Online questionnaires were administered to 34 465 individuals (aged 16 years or above) in April/May 2020 in the UK, recruited from existing cohorts or via social media. Around one-third (*n* = 12 718) of included participants had prior diagnoses of depression or anxiety and had completed pre-pandemic mental health assessments (between September 2018 and February 2020), allowing prospective investigation of symptom change.

**Results:**

Prospective symptom analyses showed small decreases in depression (PHQ-9: −0.43 points) and anxiety [generalised anxiety disorder scale – 7 items (GAD)-7: −0.33 points] and increases in PTSD (PCL-6: 0.22 points). Conversely, retrospective symptom analyses demonstrated significant large increases (PHQ-9: 2.40; GAD-7 = 1.97), with 55% reported worsening mental health since the beginning of the pandemic on a global change rating. Across both prospective and retrospective measures of symptom change, worsening depression, anxiety and PTSD symptoms were associated with prior mental health diagnoses, female gender, young age and unemployed/student status.

**Conclusions:**

We highlight the effect of prior mental health diagnoses on worsening mental health during the pandemic and confirm previously reported sociodemographic risk factors. Discrepancies between prospective and retrospective measures of changes in mental health may be related to recall bias-related underestimation of prior symptom severity.

## Introduction

The coronavirus disease 2019 (COVID-19) pandemic has resulted in a globally experienced set of interrelated stressful life events. These stressors are likely to take a toll on mental health in both the general population and those infected with severe acute respiratory syndrome coronavirus 2 (SARS-CoV-2) (Holmes et al., 2020). Stressful life events are known to heighten the risk for onset or worsening of depression, anxiety and stress-related conditions [i.e. acute/post-traumatic stress disorders (PTSD) (Daviu, Bruchas, Moghaddam, Sandi, & Beyeler, 2019; Hammen, 2005; Kilpatrick et al., 2013)]. At the same time, measures put in place to mitigate the spread of the virus diminish access to sources of support, such as social contact and routine health care (Danese & Smith, 2020; Pierce et al., 2020a), likely further contributing to the mental health burden. Establishing which groups are most adversely affected by the COVID-19 pandemic is a key research priority (Holmes et al., 2020).

Reports from longitudinal studies including pre-pandemic measures of mental health vary considerably. A UK-representative general population sample of ~53 000 adults (Pierce et al., 2020a) found elevated levels of mental distress in April 2020, compared to trends observed in 2018–2019 (12-item General Health Questionnaire). A UK birth cohort of young adults (Avon Longitudinal Study of Parents and Children; mean age 27 years, *N* = 2973) found increased anxiety, but not depression in April/May 2020, compared to 2018 (Kwong et al., 2020). A case-control sample in the Netherlands (*N* = 1517) of individuals with depression, anxiety or obsessive-compulsive disorder (OCD) found increased depression but not anxiety symptoms in April/May 2020, compared to assessments conducted between 2006 and 2016 (Pan et al., 2020). At the same time, other studies have found no observable changes in mental wellbeing, including a Dutch general population sample measuring change in anxiety and depression symptoms from November 2019 to March 2020 [*N* = 3983 (van der Velden, Contino, Das, van Loon, & Bosmans, 2020)] and a US nationally representative sample examining change in psychological distress from February 2019 to May 2020 [*N* = 1870 (Breslau et al., 2021)].

In terms of individual-level risk factors, longitudinal studies have demonstrated heightened vulnerability to worsening mental health during the pandemic among young people (Pierce et al., 2020a), females (Kwong et al., 2020; Pierce et al., 2020a) and individuals living in socio-economic adversity (Kwong et al., 2020). Several smaller studies (*n* < 500) have demonstrated worsening symptoms in groups of young people observed in the USA, Italy, India, Switzerland and China (Elmer, Mepham, & Stadtfeld, 2020; Hawes, Szenczy, Klein, Hajcak, & Nelson, 2021; Li, Cao, Leung, & Mak, 2020; Meda et al., 2021; Saraswathi et al., 2020). One study on older adults in the UK aged 55 years or over (*n* = 3281) showed an increase in reporting of mild depression and anxiety symptoms, but no change in the frequency of reporting of moderate symptoms (Creese et al., 2020). Looking at longer-term changes in mental health (April–October 2020) in a population representative sample of UK adults, worsening trajectories of mental health were more frequent among individuals from minority ethnic groups, those living in deprived neighbourhoods, those infected with SARS-CoV-2, or those experiencing financial difficulties (Pierce et al., 2021).

Prior mental health diagnoses may be an additional risk factor for worsening mental health during the pandemic. One birth cohort sample demonstrated that a history of major depressive disorder (MDD), generalised anxiety disorder (GAD) or eating disorders were associated with worsening depression and anxiety symptoms during the pandemic (Kwong et al., 2020). Trajectories of worsening mental health were shown to be more frequent among individuals with prior mental or physical health diagnoses (Pierce et al., 2021). However, a case-control study of individuals with anxiety, depression or OCD showed a different pattern of effects. Greater increases in prospectively assessed mental health symptoms were observed among individuals without pre-existing mental health diagnoses, compared to those with (Pan et al., 2020). Furthermore, in those with the most chronic and severe mental health diagnoses, slight decreases in symptom severity were observed (Pan et al., 2020). In contrast, this study also showed that individuals with prior mental health diagnoses reported a greater *perceived* impact of the pandemic on their mental wellbeing, compared to those without (Pan et al., 2020).

Perceived changes in mental health in the absence of prospectively measured symptom change may be related to memory biases widely observed in depression and anxiety (Mathews & MacLeod, 2005). Longitudinal epidemiological studies comparing prospective and retrospective diagnostic reporting suggest that approximately half of individuals reporting depressive or anxiety disorder diagnoses prospectively no longer report these diagnoses when asked to recall them at a later date (Moffitt et al., 2010). In addition, 12-month recall accuracy of depression symptoms during a clinical trial ranged from 55% to 95%, depending on the symptom (Dunlop et al., 2019). It is therefore plausible that poor recall of prior symptoms contributes to the perception of worsening mental health, even if prospective measures of symptoms show no change.

Conversely, higher levels of depression symptoms have been shown to be associated with recall of more frequent/stronger past negative mood on the PANAS-X (Wenze, Gunthert, & German, 2012) and higher current depression or anxiety symptoms were associated with overestimation of past symptom severity (Safer & Keuler, 2002). Further examination of the relationship between perceived changes in distress, retrospective estimates and prospective measurements of symptom severity, are critical to understanding vulnerabilities to worsening mental health during a pandemic.

The first aim of the current study was to examine changes in depression, anxiety and PTSD symptoms from before (September 2018–February 2020) to during (April–May 2020) the COVID-19 pandemic. To examine discrepancies across measurement approaches we investigated symptom change in three ways: (1) prospectively measured symptom change; (2) retrospectively estimated symptom change and (3) perceived changes in symptoms. Our second aim was to examine whether pre-existing mental health diagnoses and demographic factors (gender, age, ethnicity, employment status) were associated with greater change in symptoms of MDD, GAD and PTSD from before to during the pandemic.

## Methods

Data were examined from two longitudinal online studies: (1) the COVID-19 Psychiatry and Neurological Genetics study (COPING) and (2) the Repeated Assessment of Mental Health in Pandemics (RAMP; https://rampstudy.co.uk) study. COPING and RAMP administered identical questionnaires aiming to investigate symptoms of mental health conditions, neurological and respiratory health longitudinally throughout the COVID-19 pandemic (see https://osf.io/7p2ek/ for full details of questionnaires administered).

In analyses presented below, the COPING study is divided into two cohorts, ‘GLAD’ and ‘NBR’ and are compared alongside the RAMP cohort. The COPING study division was based on whether participants were existing members of the Genetic Links to Anxiety and Depression study, who had completed prospective pre-pandemic mental health assessments (GLAD cohort), or whether they were members of other NIHR BioResource studies (NBR cohort; see below). All samples were combined for regression analyses to maximise power to explore differential risk across smaller demographic groups (particularly ethnic minority groups and groups of individuals with rarer psychiatric diagnoses).

### Participants

Recruitment for COPING and RAMP was conducted on a rolling basis, beginning in April 2020. COPING participants were recruited from existing re-contactable cohorts hosted by the NIHR BioResource, the vast majority of whom (>95%) were contacted in May 2020. Participants were free to sign-up to the study at any point after receiving a study invitation, with the majority of RAMP participants completing baseline assessments in April–May 2020, and the majority of GLAD and NBR participants completing baseline assessments in May 2020.

GLAD and EDGI participants were originally recruited via social and traditional media campaigns, as well as through NHS organisations (Davies et al., 2019). IBD and general population cohorts were recruited via advertisements in blood donation centres and UK hospitals (NIHR BioResource, 2021). RAMP study participants (*n* = 8651) were recruited via social media advertising. Groups were combined for analyses (as described in the ‘Statistical analyses’ section), to maximise statistical power to detect effects related to individual difference characteristics.

Eligibility criteria for both studies were aged 16+ years and resident in the UK. GLAD eligibility criteria required either self-report of a previous depressive or anxiety disorder diagnosis, or meeting current DSM-5 criteria for depression or anxiety (Davies et al., 2019). Ethical approval was granted by: (i) NHS Health Research Authority, South West – Central Bristol Research Ethics Committee (20/SW/0078; COPING), and (ii) Psychiatry, Nursing and Midwifery Research Ethics Committee at King's College London (HR-19/20-18157; RAMP). Information sheets, consent forms and questionnaires were reviewed by the Feasibility and Acceptability Support Team for Researchers and the Service User Advisory Group.

### Measures

All questionnaire data were acquired using Qualtrics survey software (Qualtrics, Provo, UT). Participants provided consent via an online form prior to completing surveys. Demographic factors assessed were: gender, age, ethnicity and employment status (see Table 1). Lifetime mental health diagnoses were assessed using a checklist of psychiatric diagnoses (see online Supplementary materials Table S1). Diagnostic history for GLAD/EDGI participants was assessed upon sign-up to the GLAD/EDGI cohorts. For all other participants, this measure was completed at COPING or RAMP baseline assessments.

**Table 1.** Demographic details of participants across cohorts

	GLAD^a^		NBR^b^		RAMP		Overall	
	*N*	%	*N*	%	*N*	%	*N*	%
*N* contacted	36 770	–	67 783	–	n/a	–	n/a	–
*N* responded	12 718	34.6	13 096		n/a	–	n/a	–
*N* included	12 653	34.4	13 161		8651	–	34 465	–
Psychiatric diagnosis								
Depressive and anxiety disorder	9236	72.8	1119	8.5	2551	29.5	12 906	37.5
Only depressive disorder	1359	10.7	1637	12.5	1272	14.7	4268	12.4
Only anxiety disorder	840	6.6	646	4.9	798	9.2	2284	6.6
PTSD	1846	14.5	237	1.8	700	8.1	2783	8.1
OCRDs	1625	12.8	163	1.2	607	7.0	2395	7.0
Eating disorders	1380	10.9	256	2.0	482	5.6	2118	6.2
Personality disorders	950	7.5	82^c^	0.6	295	3.4	1327	3.9
Psychotic and bipolar disorder	168	1.3	8^c^	0.1	76^d^	0.9	252	0.7
Only psychotic disorders	232	1.8	29^c^	0.2	76^d^	0.9	337	1.0
Only bipolar disorders	701	5.5	67^c^	0.5	148	1.7	916	2.7
ASD	383	3.0	52^c^	0.4	191	2.2	626	1.8
ADHD	201	1.6	34^c^	0.3	114	1.3	349	1.0
No diagnosis	137	1.1	9248	70.6	2535	29.3	11 920	34.6
Gender								
Male	2271	17.9	5642	42.9	1771	20.5	9684	28.1
Female	10 106	79.9	7478	59.1	6778	78.3	24 362	70.7
Non-binary/self-defined	247	2.0	41^c^	0.3	92^c^	1.1	380	1.1
Age								
16–18	340	2.7	17^c^	0.1	697	8.1	1054	3.1
19–25	1537	12.1	308	2.3	772	8.9	2617	7.6
26–35	2850	22.5	1186	9.0	1076	12.4	5112	14.8
36–45	2453	19.4	1448	11.0	874	10.1	4775	13.9
46–55	2814	22.2	2648	20.2	1518	17.5	6980	20.3
56–65	1900	15.0	3906	29.7	2200	25.4	8006	23.2
66–70	428	3.4	1970	15.0	784	9.1	3182	9.2
71–75	230	1.8	1265	9.6	511	5.9	2006	5.8
76+	72^d^	0.6	413	3.1	209	2.4	694	2.0
Ethnicity^d^								
White	12 062	95.3	9987	75.9	8114	93.8	30 163	87.5
Asian or Asian British	98^c^	0.8	127	1.0	169	2.0	394	1.1
Black or Black British	37^c^	0.3	46^c^	0.3	76^d^	0.9	159	0.5
Mixed or multiple ethnic origins	275	2.2	121	0.9	174	2.0	570	1.7
Other ethnicity	30^c^	1.0	1^c^	0.0	70^d^	0.8	193	0.6
Employment								
Employed	3157	25.0	3414	25.9	2345	27.1	8916	25.9
Employed as key worker	5346	42.3	4437	33.7	2763	31.9	12 546	36.4
Retired	1427	11.3	4681	35.5	1908	22.1	8021	23.3
Student	672	5.3	114	0.9	720	8.3	1506	4.4
Unemployed	1103	8.7	250	1.9	500	5.8	1853	5.4

Percentages denote the proportion of individuals within a given category for each cohort (GLAD, NBR or RAMP) or in the combined sample (overall).

*Note*: GLAD and NBR combined form the COPING study.

a Pre-pandemic mental health data available.

b NBR is comprised of three sub-cohorts: the Eating Disorders Genetic Initiative (EDGI; *n* = 65); (ii) the Irritable Bowel Disease cohort (IBD; *n* = 3313) and (iii) general population cohorts (*n* = 9718).

c Note that in individual cohort analyses, these factors do not meet *a priori* power calculation criteria.

d Where available.

Depression symptoms were measured using the Patient Health Questionnaire – 9 items (PHQ-9). Each item is a diagnostic symptom of MDD and is rated on a 0 (not at all) to 3 (nearly every day) scale (score range 0–27). The PHQ-9 has a test–retest reliability of 0.84, a cut-off score of ⩾10 has 88% sensitivity and 88% specificity for major depression (Kroenke, Spitzer, & Williams, 2001), and reliable change is estimated as 7 points (Griffiths & Griffiths, 2015).

Anxiety symptoms were measured using the Generalised Anxiety Disorder scale – 7 items (GAD-7). Each item is rated on a 0 (not at all) to 3 (nearly every day) scale (score range 0–21). The GAD-7 has a test–retest reliability of 0.83, a cut-off score of ⩾10 has 89% sensitivity and 82% specificity for GAD (Spitzer, Kroenke, Williams, & Löwe, 2006), and reliable change is estimated as 5 points (Griffiths & Griffiths, 2015).

PTSD symptoms were measured using the abbreviated PTSD Checklist – 6 items (PCL-6). Each item is rated on a 1 (not at all) to 5 (extremely) scale (score range 6–30). A cut-off score of ⩾14 has 80% sensitivity and 76% specificity of 76% for PTSD (Lang & Stein, 2005), and reliable change is estimated to be 4 points (Lang et al., 2012). In the current study, the third item of the PCL-6 (‘Avoiding activities or situations because they reminded you of a stressful experience from the past’) was adapted for current (during the pandemic) symptom assessment by adding ‘this does not include activities or situations that are currently restricted or advised against’.

*Global rating of change*: After completing current symptom measures (PHQ-9 and GAD-7 only), participants provided a global rating of change, assessing perceived changes in symptoms (‘How different are these feelings to how you felt before the pandemic?’), on a 5-point scale [‘much worse’, to ‘much better’ (Robinson et al., 2017)].

*Retrospective ratings*: Indication of symptom change on global ratings of change was followed up with retrospective assessment of PHQ-9 and GAD-7 to estimate pre-pandemic symptoms (prompt: ‘thinking about how you usually felt before the pandemic…’). Retrospective scores were imputed from current scores for participants rating ‘no difference’ in symptoms.

### Assessment timepoints

#### Pre-pandemic symptoms (prospective)

GLAD cohort participants completed pre-pandemic measures of MDD, GAD and PTSD at time of enrolment into the GLAD study (September 2018–February 2020).

#### Current symptoms during the pandemic

Participants completed PHQ-9, GAD-7 and PCL-6 scales at the point of enrolment into COPING or RAMP during the pandemic (April–September 2020).

#### Pre-pandemic symptoms (retrospective)

Immediately following current symptom assessment, participants completed the global rating of change. Then, they completed retrospective estimates of pre-pandemic PHQ-9 and GAD-7 to assess recalled symptoms ‘before the pandemic’.

### Statistical analyses

Data on the PHQ-9, GAD-7 and PCL-6 were excluded if one or more items on each scale were not completed (missingness: 0.22% PHQ-9; 0.17% GAD-7; 0.41% PCL-6). There were no exclusions made based on mental health diagnostic history or demographic factors (see online Supplementary materials for power calculation).

#### Change in symptoms

We first examined perceived change in symptoms by exploring descriptive statistics for the global ratings of change measure in depression and anxiety symptoms. Next, Welch *t* tests were used to assess differences in depression, anxiety and PTSD symptom scores, as follows. Taking a *prospective* approach within the GLAD sample comparing symptoms before and during the pandemic assessing: (i) mean symptom scores, and (ii) binary threshold scores (proportions meeting clinically significant thresholds). Taking a *retrospective* approach, we compared pre-pandemic symptom scores recalled during the pandemic with current symptom scores. Finally, to examine the association between *prospective* and *retrospective* measures of depression and anxiety symptoms, correlation analyses were conducted (GLAD sample only).

#### Individual differences in symptom change

Linear regression analyses included diagnostic (mental health diagnostic history) and demographic factors as explanatory variables, entered simultaneously into multivariable regression models. The first set of analyses were restricted to the GLAD sample and examined change in depression, anxiety and PTSD symptoms by controlling for *prospectively* measured pre-pandemic symptoms. The second set of analyses were conducted in the combined sample (and each sample individually) and examined change in depression and anxiety symptoms by controlling for *retrospectively* estimated pre-pandemic symptoms. Diagnostic history variables were binary coded (present/absent), and demographic variables were coded categorically (see online Supplementary materials). Associations with current depression, anxiety and PTSD symptoms were tested in separate models.

## Results

Demographics are presented in Table 1. Frequencies of prior mental health diagnoses were 98.8% in GLAD, 29.4% in NBR and 58.1% in RAMP.

### Aim 1: describing change in symptoms

#### Perceived change in symptoms (all samples)

Overall, 55.9% reported worsening depression symptoms (15.9% much worse, 40.0% a little worse) and 54.0% reported worsening anxiety symptoms (16.7% much worse, 37.3% a little worse). In the GLAD sample, frequencies of reported worsening were 62.8% and 62.0%, for depression and anxiety symptoms, whereas, in NBR, these frequencies were 39.9% and 36.5% and, in RAMP, 70.4% and 68.9% (Table 2).

**Table 2.** Reported changes in symptoms of depression (PHQ-9) and anxiety (GAD-7) by sample (GLAD, NBR, RAMP)

Sample	Measure	Perceived change (% of sample)				
		Much worse (%)	A little worse (%)	No change (%)	A little better (%)	Much better (%)
GLAD	PHQ-9	18.1	44.7	25.3	8.2	3.5
	GAD-7	19.7	42.3	28.4	6.8	3.2
NBR	PHQ-9	6.9	33.0	56.0	2.6	1.0
	GAD-7	6.8	29.7	60.4	2.6	0.9
RAMP	PHQ-9	26.6	43.8	21.8	4.9	2.7
	GAD-7	27.4	41.5	24.7	4.2	2.3
*Overall*	PHQ-9	15.9	40.0	36.2	5.2	2.4
	GAD-7	16.7	37.3	39.7	4.5	2.1

#### Prospectively measured symptom change (GLAD only)

Comparing prospectively measured pre-pandemic symptoms to current symptoms, there was a small decrease in depression symptoms [*t*(12 110) = −8.15, *p* < 0.001, *d* = −0.07], a small decrease in anxiety symptoms [*t*(12 125) = −6.67, *p* < 0.001, *d* = −0.06] and a small increase in PTSD symptoms [*t*(11 990) = −4.30, *p* < 0.001, *d* = 0.04]. Although statistically significant, the observed changes were small in magnitude and standard deviations were large, indicating a high degree of variance. Comparing proportions of individuals meeting standardised cutoffs on each scale (⩾10 PHQ-9; ⩾10 GAD-7; ⩾14 PCL-6), there was a similar pattern of effects. Proportions of individuals meeting thresholds for ‘moderate depression’ reduced from 54.5% pre-pandemic to 51.8% [χ^2^(1, *N* = 12 098) = 17.25, *p* ⩽ 0.001] during the pandemic, for ‘moderate generalised anxiety disorder’ reduced from 40.6% to 38.9% [χ^2^(1, *N* = 12 121) = 7.83, *p* = 0.005]; and for ‘probable PTSD’ increased from 56.4% to 57.9% [χ^2^(1, *N* = 12 062) = 5.21, *p* = 0.02].

#### Retrospectively estimated symptom change (all samples)

Comparing retrospectively estimated pre-pandemic symptoms to current symptoms demonstrated significant increases in depression symptoms [*t*(34 198) = 95.86, *p* < 0.001, *d* = 0.52] and anxiety symptoms [*t*(34 323) = 86.31, *p* < 0.001, *d* = 0.47; see online Supplementary Table S2 for breakdown across samples].

#### Correlation analyses (GLAD only)

The correlations between pre-pandemic measures and retrospective estimates of PHQ-9 and GAD-7 were *r* = 0.59 [*p* < 0.001, 95% confidence interval (CI) 0.55–0.63] and *r* = 0.51 (*p* < 0.001, 95% CI 0.47–0.56), respectively. Thus, the shared variance (*r*^2^) was relatively low at 34.81% for PHQ-9 and 26.01% for GAD-7. Direct comparison of means demonstrated significantly lower scores for retrospective estimates, compared to prospective measures of both PHQ-9 [*t*(12 066) = 61.17, *p* < 0.001] and GAD-7 [*t*(12 110) = 57.16, *p* < 0.001].

### Aim 2: associations with change in symptoms

#### Prospectively measured symptom change (GLAD only)

**Fig. 1.**
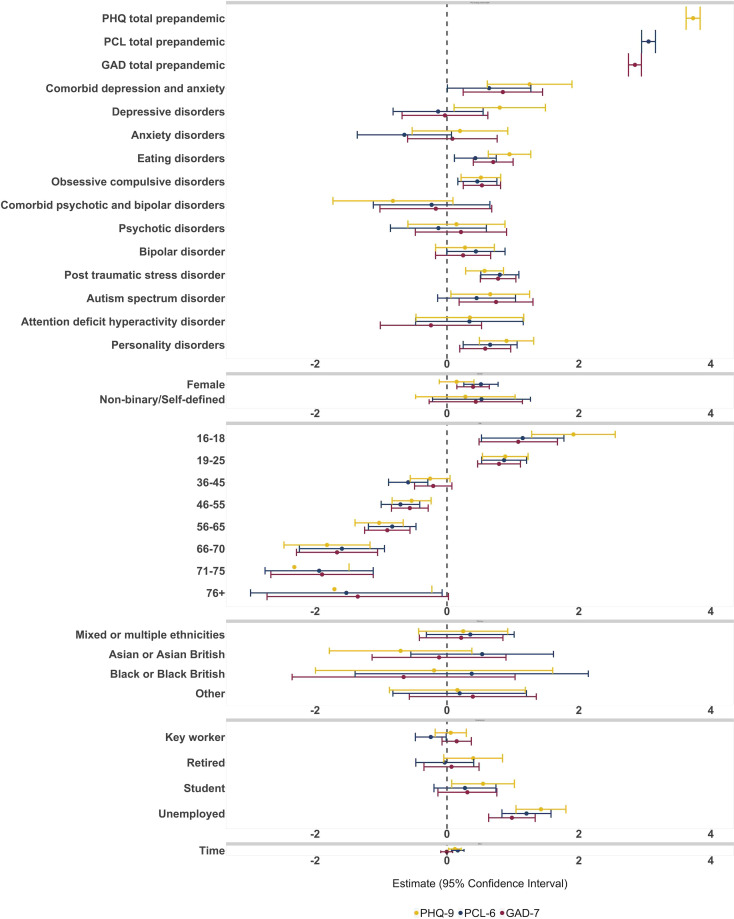
Plots detailing the effects of prior mental health diagnosis, gender, age, ethnicity and employment status on current depression, anxiety and PTSD symptoms during the pandemic, controlling for prospectively measured pre-pandemic symptom levels. Points indicate effect size estimates, error bars represent 95% CIs. Lower scores indicate a lower burden of symptoms. Note that change in symptoms was examined by including prospective prepandemic symptom measures in the model (association of prepandemic with current measures is shown in the first three data points in this figure, labelled ‘PHQ total prepandemic’, ‘PCL total prepandemic’, ‘GAD prepandemic’).

Regression analyses examined individual differences associated with change in depression, anxiety and PTSD symptoms from before to during the pandemic (Table 3). In each model, levels of pre-pandemic symptoms were positively associated with levels of current symptoms, so analyses report findings after accounting for pre-pandemic symptoms. Higher levels of current depression symptoms were associated with prior diagnosis of: (i) depression and anxiety, (ii) depression only, (iii) eating disorders, (iv) obsessive-compulsive and related disorders (OCRDs), (v) PTSD, (vi) autism spectrum disorder (ASD) and (vii) personality disorders. Higher levels of current anxiety symptoms were associated with prior diagnosis of: (i) depression and anxiety, (ii) eating disorders, (iii) OCRDs, (iv) PTSD, (v) ASD and (vi) personality disorders. Higher levels of current PTSD symptoms were associated with prior diagnosis of: (i) eating disorders, (ii) OCRDs, (iii) PTSD and (iv) personality disorders (Fig. 1).

**Table 3.** Individual differences in prospective (GLAD sample) symptom change

	Depression – prospective (PHQ-9) *n* = 12 024					Anxiety – prospective (GAD-7) *n* = 12 028					PTSD – prospective (PCL-6) *n* = 11 940				
	*B*	CI lower	CI upper	s.e.	*p*	*B*	CI lower	CI upper	s.e.	*p*	*B*	CI lower	CI upper	s.e.	*p*
Intercept	2.51	1.66	3.37	0.44	<0.001	3.00	2.20	3.80	0.41	<0.001	6.29	5.43	7.15	0.44	<0.001
Pre-pandemic symptoms^a^	0.54	0.53	0.56	0.01	<0.001	0.48	0.46	0.49	0.01	<0.001	0.50	0.48	0.52	0.01	<0.001
Psychiatric diagnoses															
Depressive and anxiety disorder	1.25	0.61	1.90	0.33	<0.001	0.85	0.25	1.45	0.31	0.006	0.64	0.01	1.27	0.32	0.05
Only depressive disorder	0.80	0.11	1.49	0.35	0.02	−0.03	−0.68	0.62	0.33	0.93	−0.13	−0.82	0.55	0.35	0.70
Only anxiety disorder	0.20	−0.53	0.92	0.37	0.59	0.08	−0.60	0.76	0.35	0.81	−0.65	−1.36	0.07	0.36	0.08
Eating disorders	0.95	0.63	1.27	0.16	<0.001	0.70	0.40	1.00	0.15	<0.001	0.43	0.11	0.75	0.16	0.008
OCRDs	0.51	0.21	0.82	0.15	<0.001	0.53	0.25	0.81	0.14	<0.001	0.46	0.16	0.76	0.15	0.002
Psychotic and bipolar disorder	−0.82	−1.73	0.09	0.46	0.08	−0.17	−1.02	0.68	0.43	0.70	−0.23	−1.12	0.65	0.45	0.61
Only psychotic disorder	0.14	−0.59	0.88	0.38	0.70	0.21	−0.48	0.90	0.35	0.55	−0.13	−0.86	0.60	0.37	0.73
Only bipolar disorder	0.27	−0.17	0.72	0.23	0.23	0.24	−0.17	0.66	0.21	0.25	0.44	0.00	0.88	0.23	0.05
PTSD	0.57	0.28	0.86	0.15	<0.001	0.78	0.51	1.04	0.14	<0.001	0.08	0.51	1.09	0.15	<0.001
ASD	0.66	0.06	1.25	0.3	0.03	0.74	0.18	1.30	0.29	0.009	0.45	−0.14	1.04	0.30	0.14
ADHD	0.35	−0.47	1.16	0.42	0.41	−0.24	−1.01	0.52	0.39	0.53	0.34	−0.48	1.16	0.42	0.41
Personality disorder	0.90	0.49	1.31	0.21	<0.001	0.58	0.19	0.97	0.20	0.003	0.65	0.25	1.06	0.21	0.002
Gender															
Female	0.15	−0.12	0.41	0.13	0.28	0.40	0.15	0.64	0.13	0.002	0.52	0.25	0.78	0.13	<0.001
Non-binary/self-defined	0.28	−0.48	1.03	0.38	0.47	0.44	−0.27	1.14	0.36	0.23	0.52	−0.22	1.27	0.38	0.17
Age															
16–18 years	1.92	1.28	2.55	0.32	<0.001	1.08	0.49	1.67	0.30	<0.001	1.15	0.52	1.77	0.32	<0.001
19–25 years	0.88	0.54	1.23	0.18	<0.001	0.79	0.46	1.11	0.17	<0.001	0.87	0.53	1.21	0.17	<0.001
36–45 years	−0.26	−0.56	0.05	0.15	0.10	−0.21	−0.49	0.07	0.14	0.15	−0.59	−0.89	−0.29	0.15	<0.001
46–55 years	−0.54	−0.83	−0.24	0.15	<0.001	−0.56	−0.84	−0.28	0.14	<0.001	−0.71	−1.00	−0.41	0.15	<0.001
56–65 years	−1.03	−1.39	−0.66	0.19	<0.001	−0.90	−1.25	−0.56	0.18	<0.001	−0.83	−1.19	−0.47	0.18	<0.001
66–70 years	−1.82	−2.47	−1.17	0.33	<0.001	−1.67	−2.28	−1.05	0.31	<0.001	−1.59	−2.24	−0.95	0.33	<0.001
71–75 years	−2.31	−3.14	−1.48	0.42	<0.001	−1.89	−2.67	−1.12	0.40	<0.001	−1.94	−2.76	−1.12	0.42	<0.001
76+ years^b^	−1.71	−3.18	−0.23	0.75	0.02	−1.35	−2.73	0.02	0.70	0.05	−1.53	−2.98	−0.07	0.74	0.04
Ethnicity															
Asian or Asian British^b^	−0.70	−1.78	0.38	0.55	0.20	−0.12	−1.14	0.90	0.52	0.82	0.53	−0.55	1.62	0.55	0.33
Black or Black British^b^	−0.20	−1.99	1.60	0.92	0.83	−0.66	−2.35	1.03	0.86	0.45	0.37	−1.39	2.14	0.90	0.68
Mixed or multiple ethnic origins	0.25	−0.43	0.92	0.34	0.48	0.22	−0.42	0.85	0.32	0.51	0.35	−0.31	1.02	0.34	0.30
Other ethnicity	0.16	−0.87	1.19	0.53	0.76	0.39	−0.57	1.35	0.49	0.42	0.19	−0.82	1.21	0.52	0.71
Employment															
Key worker	0.06	−0.18	0.29	0.12	0.64	0.15	−0.08	0.37	0.11	0.19	−0.25	−0.48	−0.01	0.12	0.04
Retired	0.40	−0.05	0.84	0.23	0.08	0.07	−0.35	0.49	0.21	0.75	−0.03	−0.47	0.41	0.22	0.88
Student	0.55	0.07	1.02	0.24	0.02	0.31	−0.14	0.76	0.23	0.17	0.27	−0.2	0.74	0.24	0.26
Unemployed	1.42	1.05	1.80	0.19	<0.001	0.98	0.63	1.34	0.18	<0.001	1.21	0.83	1.58	0.19	<0.001
Time of completion															
Survey completion date	0.08	0.01	0.14	0.03	0.01	0.00	−0.06	0.05	0.03	0.92	0.10	0.04	0.16	0.03	<0.001

*R*^2^ values for each model were: PHQ-9 = 0.02; GAD-7 = 0.00; PCL-6 = 0.01.

a Pre-pandemic symptoms varied across models – PHQ-9 scores were entered for depression model, GAD-7 scores were entered for anxiety model and PCL-6 scores were entered for PTSD model.

b Sample sizes for these effects did not meet *a priori* power criteria (based on combined sample) so effects should be interpreted with caution.

Compared to male gender, female gender was associated with higher levels of current anxiety and PTSD, but not depression symptoms. Compared to the 26–35 year old reference group: (i) 16–25 year olds reported higher current levels of depression, anxiety and PTSD symptoms; (ii) 36–45 year olds reported lower levels of PTSD symptoms; (iii) 46–75 year olds reported lower depression, anxiety and PTSD symptoms and (iv) 76+ years olds were not significantly different. Compared to White ethnicity, there were no significant effects of minority ethnic group status. Compared to being employed, being a student was associated with higher current levels of depression symptoms and being unemployed was associated with higher current levels of depression, anxiety and PTSD symptoms (Fig. 1, Table 3).

#### Retrospectively estimated symptom change (all samples)

Samples were combined to maximise power to explore differences in symptoms based on diagnostic and demographic factors (Table 4). Patterns of effects were largely consistent with the prospective model. There were three additional associations with prior mental health diagnoses: (i) prior anxiety disorder only with higher depression and anxiety symptoms; (ii) prior bipolar disorder only with higher depression symptoms and (iii) combined psychotic *and* bipolar disorder diagnosis with higher anxiety symptoms. Additionally, there were significant effects of female gender, non-binary/self-defined gender and student status on depression symptoms and key worker status on anxiety symptoms.

**Table 4.** Individual differences in and retrospective (all samples) symptom change

	Depression – retrospective (PHQ-9) *n* = 30 959					Anxiety – retrospective (GAD-7) *n* = 30 973				
	*B*	CI lower	CI upper	s.e.	*p*	*B*	CI lower	CI upper	s.e.	*p*
Intercept	4.10	3.84	4.37	0.14	<0.0001	3.64	3.39	3.88	0.13	<0.0001
Pre-pandemic symptoms^a^	0.68	0.66	0.69	0.01	<0.0001	0.66	0.65	0.67	0.01	<0.0001
Psychiatric diagnoses										
Depressive and anxiety disorder	2.68	2.53	2.83	0.07	<0.0001	2.41	2.27	2.54	0.07	<0.0001
Only depressive disorder	1.69	1.52	1.86	0.09	<0.0001	1.02	0.86	1.18	0.08	<0.0001
Only anxiety disorder	1.18	0.96	1.40	0.11	<0.0001	1.76	1.56	1.97	0.10	<0.0001
Eating disorders	0.79	0.56	1.01	0.12	<0.0001	0.55	0.34	0.76	0.11	<0.0001
OCRDs	0.46	0.24	0.67	0.11	<0.0001	0.47	0.27	0.68	0.10	<0.0001
Psychotic and bipolar disorder	0.02	−0.61	0.65	0.32	0.95	0.72	0.14	1.30	0.30	0.02
Only psychotic disorder	−0.05	−0.59	0.49	0.28	0.85	0.00	−0.50	0.50	0.25	1.00
Only bipolar disorder	0.64	0.30	0.99	0.18	<0.0001	0.32	0.00	0.64	0.16	0.05
PTSD	0.93	0.73	1.13	0.10	<0.0001	0.77	0.58	0.96	0.10	<0.0001
ASD	0.55	0.15	0.95	0.20	0.007	0.65	0.28	1.02	0.19	<0.0001
ADHD	0.34	−0.19	0.86	0.27	0.21	0.10	−0.39	0.58	0.25	0.70
Personality disorder	0.80	0.50	1.10	0.15	<0.0001	0.44	0.17	0.72	0.14	0.002
Gender										
Female	0.74	0.61	0.86	0.06	<0.0001	0.78	0.66	0.89	0.06	<0.0001
Non-binary/self-defined	0.54	0.03	1.05	0.26	0.04	0.13	−0.34	0.60	0.24	0.59
Age										
16–18 years	2.82	2.46	3.17	0.18	<0.0001	0.89	0.56	1.21	0.17	<0.0001
19–25 years	1.20	0.96	1.43	0.12	<0.0001	0.89	0.67	1.11	0.11	<0.0001
36–45 years	−0.59	−0.79	−0.40	0.10	<0.0001	−0.57	−0.75	−0.39	0.09	<0.0001
46–55 years	−0.77	−0.95	−0.59	0.09	<0.0001	0.87	−1.04	−0.70	0.09	<0.0001
56–65 years	−1.16	−1.35	−0.97	0.10	<0.0001	−1.19	−1.37	−1.01	0.09	<0.0001
66–70 years	−1.59	−1.86	−1.32	0.14	<0.0001	−1.58	−1.83	−1.33	0.13	<0.0001
71–75 years	−1.75	−2.05	−1.45	0.15	<0.0001	−1.78	−2.06	−1.50	0.14	<0.0001
76+ years	−1.77	−2.19	−1.35	0.21	<0.0001	−1.82	−2.20	−1.43	0.20	<0.0001
Ethnicity										
Asian or Asian British	0.00	−0.47	0.47	0.24	1.00	−0.19	−0.62	0.25	0.22	0.40
Black or Black British	−0.43	−1.32	0.46	0.46	0.34	−0.65	−1.48	0.18	0.42	0.12
Mixed or multiple ethnic origins	0.12	−0.28	0.52	0.20	0.55	−0.19	−0.56	0.18	0.19	0.31
Other ethnicity	0.48	−0.24	1.20	0.37	0.19	0.40	−0.27	1.07	0.34	0.24
Employment										
Key worker	0.11	−0.02	0.24	0.07	0.10	0.19	0.06	0.31	0.06	0.003
Retired	−0.19	−0.39	0.00	0.10	0.06	−0.12	−0.30	0.06	0.09	0.20
Student	0.44	0.15	0.74	0.15	0.003	0.34	0.07	0.61	0.14	0.02
Unemployed	0.65	0.40	0.90	0.13	<0.0001	0.42	0.19	0.64	0.12	<0.0001
Time of completion										
Survey completion date	−0.22	−0.25	−0.19	0.01	<0.0001	−0.22	−0.24	−0.19	0.01	<0.0001

*R*^2^ values for each model were: PHQ-9 = 0.28; GAD-7 = 0.25.

a Pre-pandemic symptoms varied across models – PHQ-9 scores were entered for depression model, GAD-7 scores were entered for anxiety model.

Results of regression analyses for individual samples (GLAD, NBR, RAMP) are presented in online Supplementary Tables S3 and S4. Overall, the direction of effects was similar across all models although some effects reached statistical significance in some samples, but not in others.

## Discussion

In a large UK study (*N* = 34 465), approximately 55% of individuals reported experiencing worsening of depression and anxiety symptoms from before to during the COVID-19 pandemic. Estimates of change in symptom severity, based on retrospective recall of pre-pandemic symptoms, indicated significant worsening of depression and anxiety symptoms from before to during the pandemic (depression *d* = 0.52, anxiety *d* = 0.49; PTSD symptoms were not assessed). However, in a subgroup (*N* = 12 718, GLAD study) who had completed prospective pre-pandemic mental health measures there were small decreases in anxiety (*d* = −0.06) and depression (*d* = −0.07) symptoms and a small increase (*d* = 0.04) in PTSD symptoms (although the size of these effects were not clinically relevant).

Even in this subgroup though, 63% reported a perceived worsening of symptoms. Exploring this effect in individuals who completed both retrospective and prospective symptom measures showed that symptoms were significantly lower in retrospective estimates of pre-pandemic symptoms than in prospective measures of pre-pandemic symptoms. This suggests that inaccurate recall of past symptom severity may contribute to the experience of perceived symptom worsening from before to during the pandemic, even in the absence of prospectively measured symptom change.

Other longitudinal studies to date have shown mixed findings regarding the mental health impact of the COVID-19 pandemic. Some studies demonstrated elevated distress, anxiety or depression (Kwong et al., 2020; Pan et al., 2020; Pierce et al., 2020a), while others showed no changes in symptoms (Breslau et al., 2021; van der Velden et al., 2020). Our findings do not show evidence of worsening symptoms of depression, anxiety or PTSD when measured prospectively, although results from retrospective measures indicate that a large proportion of individuals experienced a *perceived* worsening in their mental wellbeing, nonetheless.

Examining individual difference factors associated with risk for worsening symptoms (assessed using both prospective and retrospective measures of symptom change), we observed significant effects of lifetime diagnosis of a number of mental health conditions, female gender, young age (16–25 years) and being a student or being unemployed.

### Discrepancies across methods of estimating symptom change

Despite high levels of reported *worsening* of mental health from both global ratings of change and estimates based on retrospective recall, prospective measures indicated minor *improvements* in depression and anxiety symptoms from before to during the pandemic. Prospective measures of change in the GLAD sample indicated small (<0.5 points per scale) decreases in depression and anxiety symptoms, and a small (0.2 point) increase in PTSD symptoms. While statistically significant, these small changes in symptoms are not considered to be clinically meaningful, as reliable change is estimated to be around 7 points on the PHQ-9, 5 points on the GAD-7 (Griffiths & Griffiths, 2015) and 4 points on the PCL-6 (Lang et al., 2012).

Findings from prospective measures of change are consistent with another COVID-19 mental health study showing no significant change in depression or anxiety symptoms among individuals with prior diagnosis of depression, anxiety or OCD (Pan et al., 2020). As discussed below, various prior mental health diagnoses do appear to contribute to risk for worsening symptoms. However, small overall changes in symptoms suggest that there are as many individuals showing a decrease in symptoms as there are showing an increase from before to during the pandemic. It is plausible that for many, the stay-at-home lifestyle of the pandemic reduced daily sources of stress, such as social pressures or workplace challenges. A small overall decrease in symptoms of depression and anxiety may also be indicative of regression to the mean (Barnett, van der Pols, & Dobson, 2005) but the small increases in PTSD symptoms we observe are inconsistent with this explanation. Increase in PTSD symptoms may occur via exposure to new traumatic stressors during the pandemic (e.g. life-threatening illness such as acute COVID-19 infection, domestic/other abuse), exacerbation of existing PTSD symptoms, or may reflect broader non-trauma-related changes in mental distress. Further investigation of longitudinal PTSD symptom change in relation to experienced stressors would be important to distinguish these explanations.

The discrepancy between high levels of reported symptom worsening on global change ratings, and marginal decreases in symptom severity based on prospectively measured depression and anxiety symptom change may be explained in part by recall errors. Our findings showed that mean retrospective estimates of pre-pandemic symptom severity were significantly lower than prospective measurements of pre-pandemic symptom severity (3.11 points lower on the PHQ-9; 2.64 points lower on the GAD-7), suggesting a tendency to under-estimate past symptom severity. This tendency could plausibly contribute to an experienced ‘worsening’ in mental health, even in the absence of overall symptom change. It should be noted that while these effects were shown in both depression and anxiety symptom measures, we did not include retrospective assessments of PTSD symptoms.

Prior research examining discrepancies in prospective and retrospective reporting of mental health have shown mixed results, with individuals tending to under-report past diagnoses (Moffitt et al., 2010), but those with higher symptom levels tending to over-report recent negative mood states (Wenze et al., 2012). Other work examining discrepancies in symptom change *v.* global ratings of change over the course of a psychological intervention has also highlighted a lack of recall of past symptom endorsement [using the PHQ-9 to measure depression symptoms (Robinson et al., 2017)].

In addition to recall bias, there are other potential explanations for discrepancies in measurement observed here, as well as potential individual differences in the extent of recall bias that might be explored in future work. First, although prospective measures assessed ‘past 2 week’ symptoms, retrospective measures assessed ‘how you usually felt before the pandemic’. This likely introduced error in the timeframes being compared, as well as potential over-generalisation in retrospective reporting. Second, retrospective estimates were completed after the global change rating, which may have introduced a confirmation bias. Third, it is also possible that ratings of subjective changes in mental health capture current experiences of stress, which have previously been shown to predict longer-term changes in symptomatology (Hammen, 2005).

Prior work comparing PHQ-9 scores and global ratings of change suggested that global ratings may be a more ‘holistic’ assessment of mental wellbeing (Robinson et al., 2017). Analysis of follow-up timepoints will be critical to determine longer-term changes in mental health during the COVID-19 pandemic. Finally, a number of individual difference factors have been identified that may contribute to the extent or nature of recall biases, including depression symptom severity, personality factors (e.g. neuroticism) emotion regulation strategy use and appraisal tendencies (Levine, Lench, & Safer, 2009; Schwartz, Powell, & Rapkin, 2017). Understanding the role of these individual differences in the extent of discrepancies across measures may help to inform the types of cognitive processes contributing to effects observed.

### Risk factors: prior mental health diagnosis

Regression analyses controlling for prospectively measured pre-pandemic symptoms demonstrated that a range of pre-existing mental health diagnoses were associated with risk for worsening depression, anxiety and PTSD symptoms. These included eating disorders, OCRDs, PTSD and personality disorders. Additionally, comorbid prior depression and anxiety diagnoses and ASD diagnosis were associated with worsening depression and anxiety symptoms. Despite differing estimates of pre-pandemic symptom levels using prospective *v.* retrospective measurements, regression models controlling for retrospective estimates identified a largely similar pattern of effects. Additional significant effects, with larger effect sizes, were observed in retrospective models for ‘depression only’ and ‘anxiety only’ diagnoses and bipolar disorder (depression symptoms only), potentially due to increased power to detect these effects in a larger sample.

### Risk factors: demographic variables

We confirm prior observations demonstrating greater risk for worsening mental health among female and younger participants (Fancourt, Steptoe, & Bu, 2021; Kwong et al., 2020; Pierce et al., 2021) as well as in students and individuals who were unemployed. Female gender was associated with higher levels of anxiety and PTSD symptoms, but not depression symptoms. Younger age and being unemployed were both associated with higher levels of depression, anxiety and PTSD symptoms, while being a student was associated with higher levels of depression symptoms only. We observed no statistically significant effects of ethnicity, although it should be noted that these analyses were powered only to detect differences with a moderate effect size. Smaller effects may be present that were not observable in the present study, and examination of these should be a priority for future work. Combined factors of younger age and student/unemployment status may have put young people at particular risk of worsening mental health during the pandemic.

### Implications

Our results have two important implications. First, our findings highlight the elevated risk of worsening mental health during the COVID-19 pandemic among young people, those with prior mental health diagnoses and those who are unemployed. These groups of individuals should be prioritised for long-term mental health support during the continuing COVID-19 crisis, and beyond. Secondly, our findings highlight a known discrepancy between prospectively monitored *v.* perceived changes in symptoms of depression and anxiety (Robinson et al., 2017). Our results indicate that part of this discrepancy is related to inaccurate recall, underestimating the severity of past symptoms. Recent recommendations for evaluating the efficacy of psychological interventions highlight the need to incorporate patients' perspectives on their symptoms, as failing to do so may overlook important aspects of patient experience that may be critical for understanding treatment effects (Hobbs et al., 2021). Inclusion of patient-focused measures, such as global change ratings, in longitudinal observational research can additionally provide insight into how discrepancies in symptoms *v.* experiences change over time, and how they may contribute to important health behaviours, such as treatment-seeking.

### Limitations

Primary limitations of this work are that convenience sampling resulted in a cohort over-represented in females and individuals of White ethnicity (Pierce et al., 2020b) and that studies were conducted online only, relying on self-report measures of diagnostic history. Secondly, analyses examining prospectively measured changes in symptoms were available only in individuals with prior diagnosis of depression or anxiety (GLAD sample). The absence of an unaffected control group in these analyses may underestimate effect sizes because the presence of one diagnosis is compared not against ‘no diagnosis’, but against individuals who do not have the diagnosis of interest. However, such comparisons do provide a stringent examination of the differential risk of a range of mental health diagnoses among groups of individuals who have prior experience of mental distress. Thirdly, in GLAD it is possible to derive metrics of chronicity and current severity at the time of pre-pandemic assessments [using online questionnaires and a measure derived from the Composite International Diagnostic Interview; CIDI (Davies et al., 2019)]. However, analyses here focused on self-reported checklists of lifetime diagnoses that were available for all samples. Analyses presented therefore do not differentiate between participants whose diagnoses were current *v.* those who may have had a past diagnosis, but whose symptoms subsequently remitted. Reporting of past diagnoses was also likely to be impacted by recall errors that bias towards under-reporting. Although certainly a limitation, this would likely reduce the power to detect differences based on diagnostic status with more individuals being misallocated to the ‘no diagnosis’ group and would more likely result in false-negative than false-positive effects. Fourthly, the PCL-6 measure of PTSD symptoms does not include assessment of PTSD qualifying events. As such, this measure is not necessarily sensitive only to PTSD-related distress, and might reflect a broader range of common mental health symptoms. Additionally, regression models presented here allowed a broad exploration of sociodemographic factors associated with changes in depression, anxiety or PTSD symptoms. Future analyses might account for covariances between outcome measures, in order to gain a more detailed understanding of the unique associations between individual factors and specific symptom clusters. Finally, while the focus here on sociodemographic risk factors is informative for identifying groups at greatest risk, examination of *modifiable* cognitive or behavioural risk factors, such as emotion regulation, intolerance of uncertainty or loneliness, is essential to inform development or modification of psychological treatments to support those individuals at risk.

## Conclusion

In a large UK study combining three samples, we demonstrate that over half reported a *perceived* worsening of their mental health from before to during the COVID-19 pandemic. However, among a subsample of participants with prior depression or anxiety and pre-pandemic symptom measures, there was no evidence of clinically meaningful symptom change at the overall group level. Examining individual risk factors, prior mental health diagnosis, young age and unemployment was associated with worsening symptoms of depression, anxiety and PTSD from before to during the COVID-19 pandemic. In addition, female gender was associated with worsening symptoms of anxiety and PTSD, but not depression, and being a student was associated with worsening symptoms of depression, but not anxiety or PTSD. These groups of individuals are likely to be in particular need of support to prevent worsening of mental health during the ongoing COVID-19 crisis.

## Data Availability

Deidentified data included in analyses presented here are available from study authors on request. Analysis code will be made available at publication from: https://github.com/RAMP-COPING/PANCHANGE_analysis.

## Supplementary material

For supplementary material accompanying this paper visit https://doi.org/10.1017/S0033291722002501. click here to view supplementary material
