# Supplementary material for: Depression, anxiety and PTSD symptoms before and during the COVID-19 pandemic in the UK
Source: Psychol Med. 2022 Jul 26;53(12):5428–41. doi: 10.1017/S0033291722002501 (PMC10482709; doi:10.1017/S0033291722002501)
Supplement: Supplementary file 1 [file S0033291722002501sup001.docx]

**Supplementary materials**

## **Assessment of lifetime mental health diagnoses**

Lifetime history of prior mental health diagnoses was assessed using a checklist (Table S1) following the prompt: “Have you ever been diagnosed with one or more of the following mental health problems by a professional, even if you don't have it currently? *By professional we mean: any doctor, nurse or person with specialist training (such as a psychologist, psychiatrist etc.). Please include disorders even if you did not need treatment for them or if you did not agree with the diagnosis.”*

For two sets of diagnoses, we created separate ‘comorbid’ and ‘single diagnosis’ category variables due to high levels of correlations between diagnostic categories. These groups were depressive disorders and anxiety disorders (*r* = 0.84) and psychotic disorders and bipolar disorders (*r* = 0.71). For regression analyses, these categories were re-coded as: i) ‘comorbid depressive and anxiety disorder’, ‘only depressive disorder’ and ‘only anxiety disorder; ii) ‘comorbid psychotic and bipolar disorder’, ‘only psychotic disorder’ and ‘only bipolar disorder’.

**Table S1.** Diagnosis questionnaire response options and categorisation used in analyses

| **Individual diagnoses (response options)** | **Category** |
| --- | --- |
| Depression | Depressive disorder only |
| Depression during or after pregnancy (antenatal/postnatal depression) | Depressive disorder only |
| Premenstrual dysphoric disorder (PMDD) | Depressive disorder only |
| Anxiety, nerves or generalised anxiety disorder | Anxiety disorder only |
| Social anxiety or social phobia | Anxiety disorder only |
| Specific phobia (e.g. phobia of flying) | Anxiety disorder only |
| Agoraphobia | Anxiety disorder only |
| Panic disorder | Anxiety disorder only |
| Panic attacks | Anxiety disorder only |
| *NOTE: If both depression and anxiety were present, a separate category of comorbid depressive and anxiety disorder was created* | Depressive and anxiety disorder |
| Post-traumatic stress disorder (PTSD) | PTSD |
| Obsessive-compulsive disorder (OCD) | OCRDs |
| Body dysmorphic disorder (BDD) | OCRDs |
| Other obsessive-compulsive related disorder e.g. skin picking | OCRDs |
| Anorexia nervosa | Eating disorders |
| Atypical anorexia nervosa | Eating disorders |
| Bulimia nervosa | Eating disorders |
| Psychological overeating or binge-eating disorder | Eating disorders |
| Mania, hypomania, bipolar or manic-depression | Bipolar disorder |
| Schizophrenia | Psychosis only |
| Schizoaffective disorder | Psychosis only |
| Any other type of psychosis or psychotic illness | Psychosis only |
| *NOTE: If both bipolar and psychosis were present, a separate category of comorbid psychotic and bipolar disorder was created* | Psychotic and bipolar disorder |
| Personality disorder | Personality disorder |
| Autism, asperger's or autistic spectrum disorder | ASD |
| Attention deficit or attention deficit and hyperactivity disorder (ADD/ADHD) | ADHD |

## **Statistical analysis**

### Power calculation

We conducted a priori power calculations using G*power for t-tests (mean difference between 2 independent groups), 80% power, alpha 0.05, d = 0.3. Even group size required n = 176 per group, uneven group size (ratio 1:6) required n = 102 for the smaller group. Thus, in our analyses of combined samples presented here, there is sufficient power to examine effects of ethnicity. For consistency across models, we include ethnicity in all analyses presented, noting that for individual samples the threshold group size is not met for ethnicity.

### Variable coding details

Individual diagnoses were combined to create the following categories: i) depression *and* anxiety, ii) depression only, iii) anxiety only, iv) eating disorders, v) obsessive compulsive disorders, vi) psychotic *and* bipolar disorders, vii) psychotic disorder only, viii) bipolar disorder only, ix) post-traumatic stress disorder, x) autism spectrum disorders, xi) attention deficit hyperactivity disorder, xii) personality disorder. All demographic factors were binary coded. Age was coded categorically with binary yes/no coding in each age group. The reference categories for demographic variables in regression analyses were as follows: gender = male; age = 26-35 years (the largest age group); ethnicity = White; employment = employed.

**Table S2.** Mean (M) and standard deviation (SD) of depression (PHQ-9), anxiety (GAD-7) and PTSD (PCL-6) symptoms, and comparison (*t*-test, cohen’s *d* effect size) of using prospective prepandemic measures and retrospective prepandemic estimates, compared to baseline ‘during pandemic’ measures

|  | Baseline | | Prospective | | *Prospective  vs.  baseline* | | | *Retrospective* | | *Retrospective  vs.*  *baseline* | | |
| --- | --- | --- | --- | --- | --- | --- | --- | --- | --- | --- | --- | --- |
|  | *M* | *SD* | *M* | SD | *t* | df | *d* | *M* | *SD* | *t* | df | *d* |
| GLAD |  |  |  |  |  |  |  |  |  |  |  |  |
| PHQ-9 | 10.80 | 6.68 | 11.18 | 6.86 | -8.15* | 12110 | -.07 | 8.07 | 6.16 | 58.68* | 12561 | .52 |
| GAD-7 | 8.48 | 5.83 | 8.78 | 5.96 | -6.67* | 12125 | -.06 | 6.14 | 5.01 | 53.78* | 12596 | .48 |
| PCL-6 | 15.71 | 6.16 | 15.40 | 6.09 | -4.30* | 11990 | .04 | - | - | - | - | - |
| NBR |  |  |  |  |  |  |  |  |  |  |  |  |
| PHQ-9 | 3.31 | 4.13 | - | - | - | - | - | 2.20 | 3.25 | 45.85* | 13072 | .40 |
| GAD-7 | 2.44 | 3.66 | - | - | - | - | - | 1.65 | 2.81 | 39.05* | 13131 | .34 |
| PCL-6 | 8.54 | 3.51 | - | - | - | - | - | - | - | - | - | - |
| RAMP |  |  |  |  |  |  |  |  |  |  |  |  |
| PHQ-9 | 10.24 | 7.02 | - | - | - | - | - | 6.20 | 5.70 | 67.69* | 8563 | .73 |
| GAD-7 | 8.26 | 6.12 | - | - | - | - | - | 5.02 | 4.71 | 60.74* | 8394 | .66 |
| PCL-6 | 13.72 | 5.54 | - | - | - | - | - | - | - | - | - | - |
| Combined |  |  |  |  |  |  |  |  |  |  |  |  |
| PHQ-9 | 7.79 | 6.91 | - | - | - | - | - | 5.39 | 5.71 | 95.86* | 34198 | .52 |
| GAD-7 | 6.11 | 5.94 | - | - | - | - | - | 4.14 | 4.67 | 86.31* | 34323 | .47 |
| PCL-6 | 12.46 | 6.04 | - | - | - | - | - | - | - | - | - | - |

* denotes *p* < .001

**Results: individual cohort regression analyses**

Overall, the pattern of effects observed in analyses for each cohort individually were comparable to that observed in the combined sample. Specific differences observed are noted below.

Comparing the combined model to the individual sample models in relation to depression symptoms, there was no significant effect of: i) only anxiety disorder diagnosis in GLAD and NBR; ii) OCRDs in NBR or RAMP; iii) only bipolar disorder in GLAD and NBR; iv) ASD diagnosis in any of the individual samples (this diagnosis was significant in the combined model); v) personality disorder diagnosis in NBR. In relation to demographic variables, there was no significant effect of female gender in GLAD, non-binary/self-defined gender in GLAD or NBR, and student or unemployment status in NBR or RAMP.

Table S3. Individual differences in retrospectively estimated PHQ-9 symptom change. Estimates, confidence intervals and significance levels of pre-existing diagnostic and demographic variables (statistically significant effects are in **bold**).

|  | **GLAD** | **NBR** | **RAMP** |
| --- | --- | --- | --- |
| Intercept | **5.11 [4.36, 5.85]** | **1.87 [1.67, 2.08]** | **4.94 [4.42, 5.45]** |
| Pre-pandemic PHQ-9 | **0.61 [0.59, 0.62]** | **0.87 [0.85, 0.89]** | **0.61 [0.58, 0.63]** |
| **Psychiatric diagnoses** | |  |  |
| Depressive and anxiety disorder | **0.89 [0.23, 1.56]** | **1.15 [0.95, 1.35]** | **2.78 [2.45, 3.11]** |
| Only depressive disorder | 0.64 [-0.07, 1.36] | **0.65 [0.50, 0.79]** | **1.19 [0.83, 1.56]** |
| Only anxiety disorder | -0.46 [-1.21, 0.29] | 0.20 [-0.02, 0.42] | **0.84 [0.43, 1.26]** |
| Eating disorders | **0.91 [0.58, 1.24]** | **0.68 [0.33, 1.03]** | **0.80 [0.28, 1.32]** |
| OCRDs | **0.43 [0.12, 0.73]** | 0.08 [-0.37, 0.53] | 0.43 [-0.08, 0.93] |
| Psychotic and bipolar disorder | -0.50 [-1.42, 0.42] | -1.70 [-3.40, 0.00] | 0.01 [-1.28, 1.29] |
| Only psychotic disorder | 0.10 [-0.66, 0.87] | 0.49 [-0.49, 1.46]^$^ | -0.84 [-2.14, 0.45] |
| Only bipolar disorder | 0.40 [-0.06, 0.86] | 0.36 [-0.36, 1.09]^$^ | **1.16 [0.23, 2.09]** |
| PTSD | **0.75 [0.46, 1.04]** | **0.83 [0.45, 1.21]** | **0.89 [0.43, 1.35]** |
| ASD | 0.44 [-0.17, 1.05] | 0.10 [-0.65, 0.84]^$^ | 0.77 [-0.03, 1.56] |
| ADHD | 0.54 [-0.30, 1.38] | -0.77 [-1.70, 0.16]^$^ | -0.10 [-1.08, 0.88] |
| Personality disorder | **0.95 [0.54, 1.37]** | 0.22 [-0.45, 0.89]^$^ | **1.02 [0.30, 1.75]** |
| **Gender** |  |  |  |
| Female | 0.15 [-0.13, 0.43] | **0.40 [0.30, 0.50]** | **0.99 [0.66, 1.31]** |
| Non-binary/Self-defined | -0.19 [-0.95, 0.56] | 0.35 [-0.49, 1.18]^$^ | **1.90 [0.69, 3.11]**^$^ |
| **Age** |  |  |  |
| 16-18 years | **1.91 [1.28, 2.53]** | **3.13 [1.63, 4.63]**^$^ | **2.42 [1.79, 3.05]** |
| 19-25 years | **1.05 [0.70, 1.39]** | **0.81 [0.40, 1.22]** | **1.35 [0.81, 1.88]** |
| 36-45 years | **-0.45 [-0.76, -0.14]** | **-0.63 [-0.85, -0.41]** | **-0.57 [-1.08, -0.05]** |
| 46-55 years | **-0.47 [-0.78, -0.16]** | **-0.88 [-1.08, -0.68]** | **-0.85 [-1.31, -0.40]** |
| 56-65 years | **-1.20 [-1.59, -0.82]** | **-1.21 [-1.41, -1.01]** | **-0.98 [-1.42, -0.53]** |
| 66-70 years | **-1.66 [-2.39, -0.93]** | **-1.45 [-1.69, -1.21]** | **-1.40 [-2.01, -0.79]** |
| 71-75 years | **-2.48 [-3.42, -1.54]** | **-1.56 [-1.81, -1.30]** | **-1.53 [-2.22, -0.84]** |
| 76+ years | **-2.34 [-3.93, -0.76]**^$^ | **-1.57 [-1.90, -1.24]** | **-2.25 [-3.16, -1.34]** |
| **Ethnicity** |  |  |  |
| Asian or Asian British | -0.31 [-1.42, 0.79]^$^ | 0.06 [-0.37, 0.49] | -0.37 [-1.25, 0.51] |
| Black or Black British | 0.18 [-1.73, 2.09]^$^ | -0.25 [-0.98, 0.48]^$^ | -0.39 [-2.49, 1.71]^$^ |
| Mixed or multiple ethnic origins | 0.15 [-0.52, 0.83] | -0.15 [-0.59, 0.29] | 0.10 [-0.79, 0.98] |
| Other ethnicity | 0.21 [-0.83, 1.25] | n.r**^#^** | 0.09 [-1.31, 1.50] |
| **Employment** | |  |  |
| Key worker | 0.18 [-0.06, 0.42] | 0.07 [-0.06, 0.19] | 0.15 [-0.17, 0.46] |
| Retired | 0.33 [-0.15, 0.81] | -0.07 [-0.23, 0.08] | -0.43 [-0.87, 0.01] |
| Student | **0.59 [0.11, 1.06]** | -0.11 [-0.71, 0.49] | 0.00 [-0.56, 0.56] |
| Unemployed | **1.03 [0.65, 1.42]** | -0.24 [-0.59, 0.12] | 0.54 [-0.03, 1.11] |
| **Time of completion** | |  |  |
| Survey completion date | -0.05 [-0.16, 0.06] | -0.07 [-0.16, 0.02] | **0.45 [0.28, 0.61]** |

^#^within the NBR sample, only one individual reported Other ethnicity, this effect is not reported here; ^$^sample sizes for these effects did not meet a priori power criteria (based on combined sample) so effects should be interpreted with caution

In relation to anxiety symptoms, there was no significant effect of diagnosis of eating disorders or personality disorders in NBR and RAMP, there was no significant effect of OCRDs diagnosis in NBR, and no significant effect of ASD diagnosis in GLAD or NBR. In addition, higher symptoms of anxiety were observed in: i) 16-18 year olds in GLAD and NBR, but not RAMP; and ii) key workers, students and individuals who were unemployed in GLAD but not NBR or RAMP.

Table S4. Individual differences in retrospectively estimated GAD-7 symptom change. Estimates, confidence intervals and significance levels of pre-existing diagnostic and demographic variables.

|  | **GLAD** | **NBR** | **RAMP** |
| --- | --- | --- | --- |
| Intercept | **4.07 [3.37, 4.77]** | **1.22 [1.04, 1.40]** | **4.33 [3.86, 4.80]** |
| Pre-pandemic GAD-7 | **0.56 [0.54, 0.58]** | **0.89 [0.87, 0.91]** | **0.62 [0.59, 0.65]** |
| **Psychiatric diagnoses** |  |  |  |
| Depressive and anxiety disorder | **1.02 [0.39, 1.64]** | **1.11 [0.93, 1.29]** | **2.17 [1.87, 2.47]** |
| Only depressive disorder | -0.17 [-0.85, 0.51] | **0.40 [0.27, 0.53]** | **0.53 [0.20, 0.86]** |
| Only anxiety disorder | **0.76 [0.05, 1.46]** | **0.28 [0.08, 0.48]** | **1.61 [1.22, 1.99]** |
| Eating disorders | **0.71 [0.39, 1.02]** | **0.31 [0.00, 0.63]** | 0.42 [-0.06, 0.90] |
| OCRDs | **0.42 [0.13, 0.71]** | 0.06 [-0.34, 0.47] | **0.65 [0.19, 1.11]** |
| Psychotic and bipolar disorder | 0.42 [-0.44, 1.28] | 0.38 [-1.16, 1.91] | 0.35 [-0.83, 1.53] |
| Only psychotic disorder | 0.03 [-0.69, 0.74] | -0.02 [-0.90, 0.86]^$^ | -0.29 [-1.47, 0.88] |
| Only bipolar disorder | 0.10 [-0.34, 0.54] | **0.66 [0.01, 1.31]**^$^ | 0.63 [-0.22, 1.48] |
| PTSD | **0.79 [0.51, 1.07]** | **0.50 [0.16, 0.84]** | 0.36 [-0.06, 0.78] |
| ASD | **0.58 [0.01, 1.16]** | -0.12 [-0.80, 0.56]^$^ | **0.92 [0.19, 1.65]** |
| ADHD | 0.29 [-0.50, 1.08] | -0.49 [-1.32, 0.35]^$^ | -0.38 [-1.28, 0.52] |
| Personality disorder | **0.66 [0.27, 1.05]** | -0.23 [-0.83, 0.36]^$^ | 0.43 [-0.23, 1.09] |
| **Gender** |  |  |  |
| Female | **0.43 [0.17, 0.69]** | **0.42 [0.33, 0.51]** | **0.95 [0.65, 1.25]** |
| Non-binary/Self-defined | -0.23 [-0.95, 0.48] | 0.06 [-0.70, 0.81]^$^ | 0.68 [-0.43, 1.79]^$^ |
| **Age** |  |  |  |
| 16-18 years | **0.70 [0.11, 1.29]** | **1.98 [0.62, 3.33]**^$^ | -0.01 [-0.58, 0.57] |
| 19-25 years | **0.78 [0.45, 1.10]** | **0.85 [0.48, 1.22]** | **0.67 [0.17, 1.16]** |
| 36-45 years | **-0.42 [-0.71, -0.12]** | **-0.51 [-0.71, -0.31]** | -0.44 [-0.91, 0.04] |
| 46-55 years | **-0.63 [-0.92, -0.34]** | **-0.62 [-0.80, -0.44]** | **-1.08 [-1.49, -0.66]** |
| 56-65 years | **-1.13 [-1.50, -0.77]** | **-0.83 [-1.01, -0.65]** | **-1.39 [-1.79, -0.98]** |
| 66-70 years | **-1.63 [-2.31, -0.94]** | **-1.04 [-1.26, -0.83]** | **-1.89 [-2.45, -1.33]** |
| 71-75 years | **-2.00 [-2.88, -1.12]** | **-1.15 [-1.38, -0.92]** | **-2.31 [-2.94, -1.68]** |
| 76+ years | **-2.11 [-3.61, -0.62]**^$^ | **-1.06 [-1.36, -0.77]** | **-3.06 [-3.90, -2.23]** |
| **Ethnicity** |  |  |  |
| Asian or Asian British | 0.11 [-0.94, 1.15]^$^ | -0.16 [-0.55, 0.22] | -0.78 [-1.59, 0.02] |
| Black or Black British | -0.45 [-2.25, 1.36]^$^ | -0.05 [-0.72, 0.61]^$^ | -0.89 [-2.82, 1.04]^$^ |
| Mixed or multiple ethnic origins | -0.25 [-0.88, 0.39] | -0.25 [-0.65, 0.15] | -0.23 [-1.04, 0.58] |
| Other ethnicity | 0.11 [-0.88, 1.10] | n.r**^#^** | 0.06 [-1.22, 1.33] |
| **Employment** |  |  |  |
| Key worker | **0.41 [0.18, 0.64]** | 0.04 [-0.07, 0.16] | 0.10 [-0.18, 0.39] |
| Retired | 0.21 [-0.24, 0.67] | 0.02 [-0.12, 0.16] | -0.37 [-0.77, 0.04] |
| Student | **0.64 [0.19, 1.09]** | 0.23 [-0.31, 0.77] | -0.29 [-0.80, 0.22] |
| Unemployed | **0.87 [0.51, 1.23]** | -0.17 [-0.49, 0.15] | -0.06 [-0.57, 0.46] |
| **Time of completion** |  |  |  |
| Survey completion date | -0.08 [-0.19, 0.02] | -0.07 [-0.15, 0.02] | **0.16 [0.02, 0.31]** |

^#^within the NBR sample, only one individual reported Other ethnicity, this effect is not reported here; ^$^sample sizes for these effects did not meet a priori power criteria (based on combined sample) so effects should be interpreted with caution
